# Self-reported allergies in Russia and impact on skin

**DOI:** 10.1177/2050312120957916

**Published:** 2020-09-12

**Authors:** Sophie Seité, Charles Taieb, Tamara Lazic Strugar, Peter Lio, Elena E Bobrova

**Affiliations:** 1 La Roche-Posay Dermatological Laboratories, Levallois-Perret, France; 2 European Market Maintenance Assessment, Fontenay-sous-Bois, France; 3 Icahn School of Medicine at Mount Sinai, New York, USA; 4 Medical Dermatology Associates of Chicago, Chicago, IL, USA; 5 Department of Allergology and Immunology Saint-Petersburg State Medical University, Saint-Petersburg, Russia

**Keywords:** Allergies, food allergy, skin allergy, respiratory allergy, prevalence, skin side effects

## Abstract

**Introduction:**

The rising prevalence of allergies can substantially impact the skin, which is one of the largest targets for allergic and immunologic responses.

We present the results of an online survey assessing self-reported allergy prevalence in Russians, outline the populations who report allergies and characterize the skin conditions associated with allergy.

**Methods:**

An online survey was conducted in Russia of 2010 adults as a representative sample of the general Russian population.

**Results:**

A total of 34.9% of Russian adults (mean age: 41.3 ± 14.4 years old) reported having allergies. Reported allergies included skin allergies (73.3%), food allergies (53.9%) and respiratory allergies (43.4%), and 65.9% reported their allergies had been diagnosed by a doctor. In total, 75.1% of those who reported allergies also reported experiencing associated skin reactions, they were 1.5–5.5 times more likely to report a cutaneous disease and were 1.5 times to report sensitive skin compared to those who did not report allergies. In addition, those that reported allergies were also 2 times more likely to report experiencing skin reactions when using skincare products.

**Conclusion:**

It is estimated that over 35 million Russian adults have allergies. These results will help raise awareness about the burden of allergies and the need to develop solutions to mitigate their impact on health.

## Introduction

About one-third of the world’s population suffers from an allergic disease.^1^ In the Western world, prevalence of allergies, including hay fever, asthma and especially now food allergy, has been on the rise, a phenomenon referred to as the “allergy epidemic.”^1^ The most common chronic allergic diseases include allergic rhinitis, allergic conjunctivitis, bronchial asthma and atopic dermatitis. The chronic course of allergies affects every sphere of human life. Furthermore, care for allergic patients is associated with high healthcare costs. Therefore, high quality and rational therapy of allergic conditions is very important for preventing relapse and improving the patient’s quality of life.

According to recent statistics, the prevalence of allergies in the adult population is close to 30%, while in the pediatric population it is almost 50%.^2^ The incidence of all forms of allergies has been universally on the rise. The highest increase is reported for asthma, allergic rhinitis, food and drug allergies. According to official figures, the incidence of allergy in Russia ranges from 5% to 20.5%, while according to research conducted by the Institute of Immunology of the Federal Medical and Biological Agency, Russia, these figures range between 17.5% and 30%.^3^

A study of the rising incidence of allergies from 1990 to 1999 in Russia shows a 10% growth in the overall incidence for all types. For example, the prevalence of allergies in the northwestern region of Russia between 1990 and 1996 was 13.9%.^4^

As part of the GA2LEN epidemiological study (2009) at the Institute of Immunology of the Federal Medical and Biological Agency, patients treated by an allergist were asked to fill in a questionnaire. Overall, 550 patients responded. Among them, 39 respondents had an established diagnosis of asthma, while 84 patients had suspected asthma. While 121 patients had sinusitis, 56 had a combination of sinusitis and asthma. Moreover, 253 patients had eczema or skin allergy, 11 had a hypersensitivity to non-steroidal anti-inflammatory drugs, 144 suffered from a food allergy.^5^

The global prevalence of atopic dermatitis is rising every year, particularly in the pediatric population: based on the latest estimates, 15%–30% of children and 2%–10% of adults are affected. In 45% of children with atopic dermatitis, the condition emerges at the age of 0–6 months, in 60% within the first year of life, and in 85% in the first 5 years of life.^6^ Here, we describe the results of an online survey assessing self-reported allergy prevalence in Russia, outline the populations who report allergies and characterize the skin conditions associated with allergy.

## Methods

### Study population

**Figure 1.**
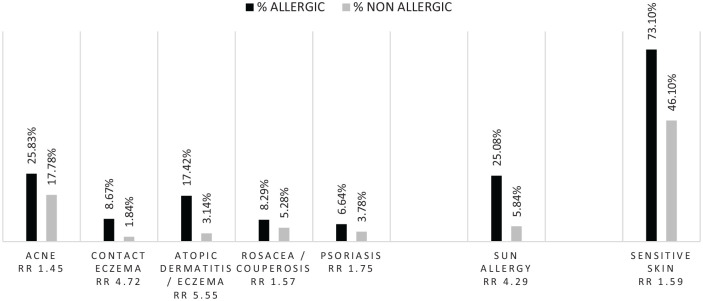
Skin diseases in the two populations.

A polling institute (HC Conseil, Paris, France) conducted an online survey in Russia between November 2018 and January 2019 among 2010 individuals (age: 18 years and older), a representative sample from the general adult Russian population. A sample size of 2000 participants was calculated in order to approach the “prevalence” of allergy, with a margin of error of ±2% (at 95% risk) based on an assumption of 30%. The participants were selected using a stratified random sampling method from a database of Internet users who have agreed to participate in various panel surveys. Fixed quotas of individuals fulfilling predefined socio-demographic criteria were recruited. Drawing on national population data, these quotas were based on the following aspects: sex, age, socio-professional status and regional distribution, thereby ensuring accurate representation of the Russian sample population (Figure 1).

### Survey

Because this study used completely anonymized data and did not involve patient contact, an institutional review board approval was not required. We are in a self-reported methodology, the diagnoses haven’t been confirmed in all cases by a doctor. Respondents were asked a range of socio-demographic questions including gender, age, occupation/social class, area of residence; tobacco use; Fitzpatrick classification phototype; presence of allergies; type of allergies; allergens; medical diagnosis confirmation; therapeutic treatment; symptoms, skin pathologies, skin effects and skin symptoms. Questions regarding the impact of environmental factors like exposure to environmental pollution and sun were also asked. The survey questionnaire has been developed with allergists and dermatologists (Supplementary Table).

### Statistical analysis

In this descriptive study, participants who reported allergies were compared to participants who did not report any allergies. Quantitative variables were expressed as mean and standard deviation. Qualitative variables were expressed as frequencies and percentages. Comparisons between groups were performed using the Student test in the case of quantitative variables; for categorical variables, intergroup comparisons were done with the χ^2^ test. Relative risk (RR) was calculated for comparison of the population who reported allergies to the population who did not reported allergies. The level of significance was set at 5%. Statistical analyses were performed using R software version 3.6.1.

## Results

### General population

Out of the 2010 respondents (18–74 years old, 46.8% males and 53.2% females), 34.9% of subjects (mean age: 41.7 ± 14.4 years old) self-reported having allergies (of which 37.6% were men and 62.4% were women). In total, 4.6% of the total population lived in rural areas (<3000 inhabitants), 10.1% in suburban or medium size cities (between 3000 and 100,000 inhabitants), 85.2% in large cities (>100,000 inhabitants) and 38.3% were smokers. The phototype repartition of the total population was 17.4%, 39.9%, 32.8%, 8.2%, 1.5% and 0.1% for, respectively, phototype I–VI.

Reported allergies included skin allergies (73.3%), food allergies (53.9%) and respiratory allergies (43.4%). A total of 65.9% reported their allergies had been diagnosed by at least one doctor, a dermatologist or an allergist most frequently (Table 1).

**Table 1.** Doctors who diagnosed allergies.

	n	%
Participants reporting an allergy	702	34.93
Participants able to name the allergy	308	43.87
Percentage of participants diagnosed by a doctor	463	65.95
Health professional who diagnosed the participant’s allergy		
Dermatologist	197	42.55
Allergy specialist	164	35.42
General practitioner	68	14.69
ENT doctor	10	2.16
Pulmonary specialist	8	1.73
Pediatrician	7	1.51
Other specialized physician	7	1.51
Acupuncturist	1	0.22
Homeopathic doctor	1	0.22

However, many reported not using any treatment (corticosteroids, antihistamine or other)—respectively, 37.5%, 52.1% and 39% of those with skin, food and respiratory allergies.

A total of 43.9% were able to identify the allergen(s) responsible for their allergies (mainly food and pollens), as well as the main symptoms associated with their allergies were allergic rhinitis or eczema (Table 2).

**Table 2.** Symptoms and allergens related by the allergic population.

Symptoms associated with allergy reported by participants	n	%
Allergic rhinitis (hay fever)	294	41.88
Eczema/atopic dermatitis	236	33.62
Edema	171	24.36
Conjunctivitis	139	19.80
Other	134	19.09
Asthma	58	8.26
Bronchitis with wheezing	56	7.98
Allergen reported by participants	n	%
Food allergens	284	40.46
Pollens	282	40.17
Other	195	27.78
Dogs, cats, ferrets, other animals	98	13.96
Mold	84	11.97
Dust mites	54	7.69
Latex	28	3.99
Hymenoptera (bees, wasps, hornets, etc.)	26	3.70
Cockroaches	5	0.71

In total, 75.1% of those who reported allergies also reported experiencing associated skin reactions. In 52.9%, a doctor diagnosed this skin reaction, and 42.5% of those with skin reaction reported resorting to topical and/or oral treatments (Table 3).

**Table 3.** Skin reactions associated with allergies.

	n	%
Percentage of participants reporting skin reaction	527	75.07
Percentage managed by a doctor	279	52.94
Health professional who managed the skin reaction?		
Dermatologist	185	66.31
Allergy specialist	68	24.37
General practitioner	15	5.38
Participants reporting prescribed treatment for skin reaction	224	80.29
What kind of treatment was prescribed for your skin reaction?		
Topical	164	73.21
Oral	121	54.02
Dermocosmetic	53	23.66

### Allergic population versus non-allergic population

The population who reported allergies included more women (62.4% vs 48.2%) (p < 0.0001) in comparison to the population who did not report allergies. However, the two populations were similar in their geographical location, age phototype (light (I, II, III) versus dark skin (IV, V and VI) (90.9% and 9.1% vs 89.8% and 10.2%, respectively) or their smoking status (37.2% vs 38.9%).

Those who reported allergies were 1.5–6 times more likely to also report a skin disease (atopic dermatitis (RR = 5.55 (3.9–7.87), p < 0.001), sun allergy (RR = 4.29 (3.31–5.55), p < 0.001), contact eczema (RR = 4.72 (2.93–7.57), p < 0.001), psoriasis (RR = 1.75 (1.18–2.60), p < 0.001), acne (RR = 1.45 (1.21–1.73), p < 0.01) or rosacea (RR = 1.57 (1.1–2.23), p < 0.02)) and were 1.5 times more likely to report sensitive skin (RR = 1.58 (1.47–1.70), p < 0.001) compared to those who did not report allergies (Figure 1).

They were significantly more likely to report sensitive skin (72.2% vs 44.4%, p < 0.0001), particularly very sensitive skin (13.7% vs 6.3%, p < 0.001) but also sensitive eyes (67.9% vs 55.1%, p < 0.001) and having parents with sensitive skin (37.2% vs 20.2%, p < 0.002). Interestingly, 29.3% of those who reported allergies also reported having atopic dermatitis during childhood versus 7.3% for those who did not report allergies (p < 0.0001).

**Figure 2.**
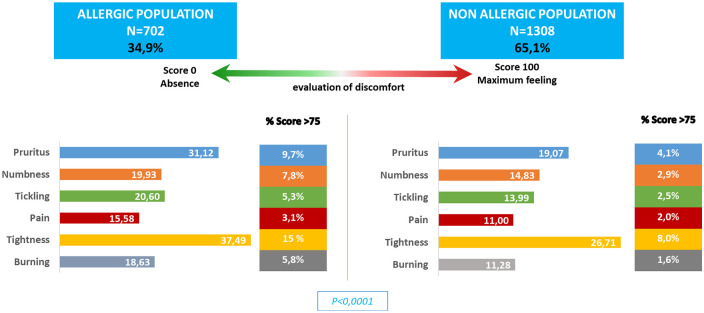
Skin discomforts in the two populations.

**Figure 3.**
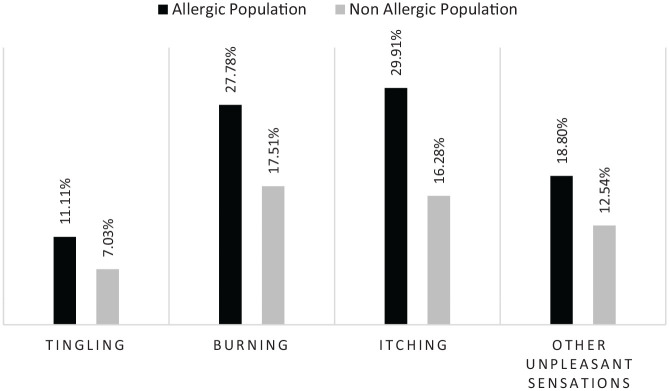
Skin discomforts associated with skincare products in the two populations.

Those who reported allergies were more likely to experience skin discomfort and reported a higher incidence of severe skin discomfort (Figure 2). They were also more likely to report experiencing skin reactions (pruritus: RR = 1.84; burning: RR = 1.59 or tickling: RR = 1.58 p < 0.001) when using skincare products (Figure 3).

### Environmental impact

The population who reported allergies was significantly more impacted by air, water, ground, noise, light and radiation pollution (p < 0.04) than the population who did not report allergies (Table 4).

**Table 4.** Impact of the pollution in the two populations.

	Impacted				Worried		
	Allergic n = 748	Non allergic n = 1255	RR	P-value	Allergic n = 748	Non allergic n = 1255	RR
Air	89.97%	88.84%	1.01	<0.05	43.58%	43.59%	1.00
Water	41.31%	34.10%	1.21	<0.05	7.09%	7.17%	0.99
Soil	30.08%	25.10%	1.20	<0.05	3.07%	2.71%	1.13
Noise	65.24%	58.41%	1.12	ns	28.07%	27.81%	1.01
Light	49.47%	38.49%	1.29	<0.05	11.76%	8.84%	1.33
Radiation	26.87%	20.80%	1.29	ns	5.08%	5.18%	0.98

They more frequently claimed that pollution affected their way of life (81.6% vs 70.9%, p < 0.0001) and had a health and well-being impact (94.4% vs 87.6%, p < 0.0001). They also more commonly noted an impact of pollution on their skin (65.2% vs 47.4%, p < 0.001, quite to very important for 65.8% vs 45.9%, p < 0.0001) and use of dermocosmetics to protect their skin against pollution (47.5% vs 40.5%, NS).

In the population who reported allergies, slightly less had moderate and intense daylight solar exposure than the population who did not report allergies (74.4% vs 62.4%, p < 0.001). In addition, only 17.1% reported not using any photoprotection in comparison to 23.4% in the population who did not report allergies (p < 0.0001) and they were more likely to apply it during outdoor leisure activity (54.1% vs 52.8%, NS) or when working outdoors (40.6% vs 37.5%, NS) but applied sunscreen similarly during intense sun exposure (78.7% vs 75.2%, p < 0.05).

## Discussion

In this survey of a representative sample of the general Russian population, 34.9% of survey respondents reported having allergies. Self-report may be one limitation of this study, as only 65.9% of the respondents who reported allergies said that these allergies had been officially diagnosed by a doctor. This can be problematic because a non-immunologically based adverse response to a food may easily be misconstrued to be an allergic reaction and self-reported as such.^7^ Another example is for sun allergy reported as an allergy during this survey. Other limitations of this study are first that only adults 18 years and older were sampled when allergy rates are increasing most rapidly among children,^8^ second that the questionnaire used has not been previously validated and third that the Russian population with no Internet access was excluded and that participants were selected from a group who have consented to market research. There are many theories attempting to explain the ongoing escalation in allergy prevalence. The role of the skin barrier in allergic sensitization has been well-described. Specifically, dysfunction of the skin barrier can increase the likelihood of allergens coming into contact with the immune system, which can trigger sensitization.^9^ However, the impact of allergies on other skin conditions has been less thoroughly characterized. Nonetheless, the survey results presented here show a clear association between reporting any type of allergy and reporting a skin disease or skin sensitivity. While some of these links are relatively well established, such as that between food allergy and atopic dermatitis, others are less clear.^10^ Interestingly, elderly atopic dermatitis (more numerous in polluted environment)^11^ that is increasingly impacting in society must be taken into consideration in our results because in the elderly population skin prick tests revealed a positivity for aeroallergens in 49.6% of patients and most of them being polysensitized (55%). In addition, food skin prick tests were positive in 25% of patients.^12^ In addition, a recent publication classify the cutaneous manifestations in patients with non-celiac gluten sensitivity and wheat allergy.^13^

Our results indicate that the population who reported allergies was similar for its geographical location or its smoking status than the population who did not report allergies. Nevertheless, the population who reported allergies was significantly more impacted by air pollution or water or light and radiation pollution than the population who did not report allergies. This could indicate that perhaps pollution do not induced first allergy but that people with allergy are more sensitive and could be more easily sensitized to other allergens. Furthermore, some of the symptoms described in this survey have been demonstrated to be enhanced by the contact of chemicals and especially by air pollutants from in- and/or out-door origin.^14^

Last but not least, the lifestyle burden on people who suffer from allergies is significant. Anxiety, impact on relationships, embarrassment and frequent interruptions to normal tasks brought on by respiratory, food and skin allergy symptoms all contribute to poorer quality of life in those with allergies.^15–18^

## Conclusion

Understanding allergy is critical to providing care to the vast proportion of Russians who suffer from its symptoms. Much work still needs to be done in developing ways to manage allergies. Strategies such as avoidance can be an option as 43.9% of survey respondents were able to identify the causative allergens. Furthermore, external environmental factors can disrupt the skin barrier, thus increasing the risk of development of dermatological diseases. In order to heal the dysfunctional skin barrier, it is important to replete the deficiency of moisturizing factors and to calm sensitive and allergic skin with use of proper skin care products. Without well-developed therapeutic solutions to existing allergies, the prevalence of allergies is bound to continue to rise, even as incidence stabilizes.

## Supplemental Material

Supplementary_Table_1 – Supplemental material for Self-reported allergies in Russia and impact on skin Click here for additional data file. Supplemental material, Supplementary_Table_1 for Self-reported allergies in Russia and impact on skin by Sophie Seité, Charles Taieb, Tamara Lazic Strugar, Peter Lio and Elena E Bobrova in SAGE Open Medicine
