# Supplementary material for: Self-reported allergies in Russia and impact on skin
Source: SAGE Open Med. 2020 Sep 12;8:2050312120957916. doi: 10.1177/2050312120957916 (PMC7488871; doi:10.1177/2050312120957916)
Supplement: Supplementary_Table_1 – Supplemental material for Self-reported allergies in Russia and impact on skin [file Supplementary_Table_1.pdf]

Supplementary Table: Survey questionnaire

- Do you have any allergies?

1. Yes
2. No

- Do you have or have you had a food allergy?

1. Yes
2. No

- a respiratory allergy (rhinitis or asthma)?

1. Yes
2. No

- a skin allergy?

1. Yes
2. No

Does your allergy cause your skin to react?

1. Yes
2. No

Has a doctor diagnosed your allergy?

1. Yes
2. No

-1 was it a...

Ask if code 1 in Q

general practitioner
dermatologist
allergy specialist
pulmonary specialist
ENT doctor
pediatrician
acupuncturist
homeopathic doctor
other specialized physician

- Do you know what your allergy is called?

1. Yes
2. No

- What are the symptoms of your allergy?

asthma
allergic rhinitis (hay fever)
bronchitis with wheezing
eczema/atopic dermatitis
conjunctivitis
edema
other

- Do you know the allergen?

dust mites
cockroaches
pollens
food allergens
dogs, cats, ferrets, other animals
mold
Hymenoptera (bees, wasps, hornets etc.)
latex
other

- Have you received treatment for your food allergy?

Ask if code 1 in Q

1. Yes
2. No

-1 - Was it a corticosteroid?

no
yes, for topical use (cream or other)
yes, oral form (tablets or other)

-2 - Was it an antihistamine?

no
yes, for topical use (cream or other)
yes, oral form (tablets or other)

-3 - Was it another drug (including plant-based medicinal preparations)?

no
yes, for topical use (cream or other)
yes, oral form (tablets or other)

- Have you received treatment for your respiratory allergy?

Ask if code 1 in Q 53

1. Yes
2. No

-1 - Was it a corticosteroid?

no

yes, for topical use (cream or other)

yes, oral form (tablets or other)

-2 - Was it an antihistamine?

no

yes, for topical use (cream or other)

yes, oral form (tablets or other)

-3 - Was it another drug (including plant-based medicinal preparations)?

no

yes, for topical use (cream or other)

yes, oral form (tablets or other)

- Have you received treatment for your skin allergy?

Ask if code 1 in Q 54

1. Yes
2. No

-1 - Was it a corticosteroid?

no

yes, for topical use (cream or other)

yes, oral form (tablets or other)

-2 - Was it an antihistamine?

no

yes, for topical use (cream or other)

yes, oral form (tablets or other)

-3 - Was it another drug (including plant-based medicinal preparations)?

no

yes, for topical use (cream or other)

yes, oral form (tablets or other)

-4 - Was it over-the-counter dermocosmetic products (soap, lotion or cream for the face or body)

1. Yes
2. No

-Did you consult a health care professional to manage the skin reactions?

Ask if code 1 in Q 55

1. Yes
2. No

-1 - Which health care professional(s)?

general practitioner
dermatologist
allergy specialist
pediatrician
homeopathic doctor
acupuncturist
pharmacist
nurse
other

-2 - Did you receive treatment for your skin reaction?

1. Yes
2. No

-3 - Which treatment did you receive for your skin reaction?

a drug taken orally

a drug for topical use (cream or lotion or medicinal preparation)

a cosmetic: over-the-counter soap, lotion or cream for the face or body
